# Impact of prophylactic TNF blockade in the dual PD-1 and CTLA-4 immunotherapy efficacy and toxicity

**DOI:** 10.15698/cst2019.07.193

**Published:** 2019-06-27

**Authors:** Maite Alvarez, Itziar Otano, Luna Minute, Maria Carmen Ochoa, Elisabeth Perez-Ruiz, Ignacio Melero, Pedro Berraondo

**Affiliations:** 1 Program of Immunology and Immunotherapy, CIMA Universidad de Navarra, Pamplona, Spain.; 2 Navarra Institute for Health Research (IDISNA), Pamplona, Spain.; 3 Centro de Investigación Biomédica en Red de Cáncer (CIBERONC), Madrid, Spain.; 4 Department of Oncology, Hospital Costa del Sol, Marbella, Spain.; 5 Department of Oncology, Clínica Universidad de Navarra, Pamplona, Spain.; 6 Department of Immunology and Immunotherapy, Clínica Universidad de Navarra, Pamplona, Spain.

**Keywords:** CTLA-4, PD-1, immunotherapy, cancer, tumor necrosis factor, toxicity, colitis

## Abstract

The TNF blockade therapy is currently a well-established treatment option for a variety of autoimmune diseases such as rheumatoid arthritis (RA), psoriasis or Crohn's disease, given the proinflammatory role of TNF in the course of these diseases. Importantly, TNF neutralization is also used for the treatment of corticosteroid-refractory immune-related adverse events (irAEs) induced by the combined anti-PD-1 and anti-CTLA-4 immunotherapy. The manifestation of these toxicities is an important limiting factor for the successful implementation of the inhibitory checkpoint blockade therapy (ICB), restraining its anti-tumor efficacy. In our recent study (Perez-Ruiz *et al.*, Nature 569(7756): 428-432.), we analyzed the potential impact of prophylactic TNF neutralization therapy in the anti-PD1/CTLA-4 efficacy. Through several mouse models, we demonstrated that TNF neutralization ameliorated ICB-exacerbated colitis in addition to improving ICB-dependent anti-tumor efficacy. Similar results were obtained after prophylactic TNF blockade in graft vs host xenografted mouse models with human immune cells, which showed a reduction in colitis and hepatitis. Importantly, there was a preservation of the immunotherapeutic control of xenografted tumors after ICB treatment. Moreover, TNF and TNF-dependent gene expression is upregulated in the colon mucosa from patients affected by colitis as a side effect of ipilimumab and nivolumab. Our results, thus, provide evidence of the successful combination of prophylactic TNF neutralization with ICB therapy strategy to ameliorate toxicities, while keeping or even ameliorating anti-tumor efficacy. The prophylactic TNF blockade strategy is clinically feasible since excellent TNF inhibitors have been approved for the treatment of autoimmunity and are used for the immune-related serious adverse events in immunotherapy.

Inhibitory checkpoint blockade (ICB) immunotherapy has produced very significant advances in the fight against cancer (**Figure 1**). The synergistic combination of PD-1 and CTLA-4 blockade therapy has been successfully used against a variety of tumors such as melanoma, renal cell carcinoma, or non-small-cell lung cancer with reportedly improved results when compared to single ICB therapy (**Figure 1A-C**). Unfortunately, the appearance of serious immune-related adverse events (irAEs) has hindered the overall anti-tumor response of these therapies by forcing a reduction of the dose or, in over 30% of patients, discontinuation of the treatment (**Figure 1C**). The course of action towards these irAEs consist of steroids treatment followed by transient or permanent treatment discontinuation and TNF blockade therapy is routinely used when there is no response to steroids. By using tumor-transplanted mouse models and xenograft mouse models, we have demonstrated that prophylactic blockade of TNF right before starting the dual anti-PD1 and anti-CTLA-4 therapy prevents irAEs, while maintaining or even enhancing to some extent the therapeutic efficacy of combined ICB therapy (**Figure 1D**).

**Figure 1:**
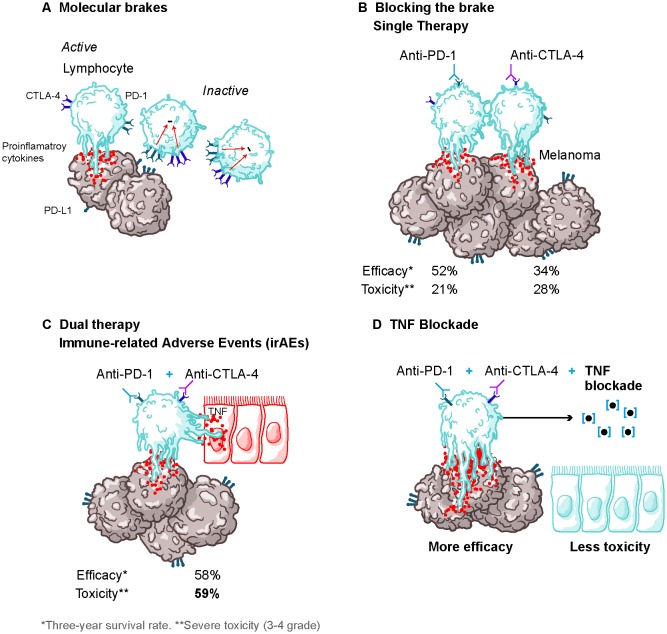
FIGURE 1: Reducing the toxicity of cancer immunotherapy. **(A)** The presence on activated lymphocytes of the inhibitory receptors PD-1, which binds to PD-L1, and CTLA-4, which binds to the costimulatory molecules CD80 and CD86, causes immune cell dysfunction facilitating tumor evasion. **(B)** The introduction of inhibitory blockade therapy that prevents PD-1/PD-L1 or CTLA-4/CD86-CD80 interactions by monoclonal antibody therapy has shown to improve CD8 T cell cytotoxic function and thus has been successfully implemented in a variety of tumors such as melanoma, with little toxicity. **(C)** Dual therapy that combines anti-PD-1 and anti-CTLA-4 has a more effective anti-tumor response when compared to single therapy, but, at the cost of the increase in the frequency of immune-related adverse events, which induces tissue damage, being TNF an important mediator of this effect. **(D)** The administration of dual ICB therapy in combination with prophylactic TNF neutralization is able to palliate the treatment-related toxicity side effects while becoming more efficacious towards the anti-tumor response by limiting the activation induced cell death (AICD) on the lymphocytes.

The role of the TNF in the induction of ICB-mediated irAEs was proven in an autoimmune colitis mouse model that involved the induction of inflammatory bowel disease by dextran sulfate sodium (DSS). In this model, ICB therapy worsened the DSS-elicited autoimmune colitis with marked thickening of the intestinal wall and immune cell infiltration into the colon mucosa, signs that were prevented when TNF was neutralized by anti-TNF antibodies or etanercept. Importantly, the blockade of TNF resulted in an improvement of tumor survival in DSS-treated tumor-bearing mice after ICB therapy, unlike IL-6 neutralization, which harmed rather than improved ICB-enhanced anti-tumor responses. In the absence of DSS-induced colitis, ICB efficacy was retained when anti-TNF or etanercept was co-administered.

The effectiveness of prophylactic TNF neutralization treatment in ICB therapy was linked to a higher abundance of infiltrated antigen-specific CD8^+^ T cells in the tumor microenvironment and tumor-draining lymph nodes (dLN) with the presence of gp70- and OVA-positive CD8^+^ T cells in MC38 and B16-OVA mouse models, respectively. Phenotypical analysis of intratumoral antigen-specific CD8^+^ T cells using a non-competing monoclonal antibody against PD-1 showed that these cells had a lower surface expression of this surface marker when mice received ICB therapy. Such downregulation of PD1 was also deadly observed when anti-TNF or etanercept was used. Interestingly, high levels of PD-1 have been correlated, along with other markers, with an exhaustion phenotype that it is characterized by functional impairment in CD8 T cells. These results raised the possibility that TNF-dependent ICB improved efficacy could be the result of a reduction in the proportion of antigen-specific CD8^+^ T cells that display an exhaustion phenotype. However, when the expression of various exhaustion markers (Tim3, Lag3, CTLA-4, BTLA, CD160, and 2B4) was analyzed, no differences were observed in any of them when compared to DSS-control treated antigen-specific CD8^+^ T cells independently of TNF neutralization. These data can be explained by a selective expansion of PD1^-/low^ CD8^+^ T cells as a result of ICB treatment.

Another important difference observed was a reduction in the proportion of cell death within total and antigen-specific CD8^+^ T cells in the tumor microenvironment and dLN of mice as a result of systemic TNF neutralization, suggesting an attenuation of the activation-induced cell death (AICD). A similar reduction in apoptosis was obtained when T cell-receptor (TCR)-transgenic mouse CD8^+^ OT-I and Pmel-1 cells were activated *in vitro* with their corresponding peptides and co-cultured with anti-PD1/CTLA-4 in the presence of anti-TNF or etanercept in comparison with ICB treatment alone. Furthermore, the induction of AICD by the *in vitro* activation of human CD8^+^ T cells from healthy donors with anti-CD3/CD28 was reduced by infliximab or etanercept treatment. Thus, these data suggest that the mechanisms of action for TNF blockade seem to operate through a reduction of ICB-induced AICD. This immune phenomenon has been recently reported to limit the anti-tumor efficacy of the anti-PD-1/anti-CTLA-4 combined treatment in a low tumor burden setting characterized by tumor infiltration with partially exhausted T lymphocytes. In this report, IFN-γ was identified as a critical mediator of AICD, but IFN-γ blockade will likely affect the antitumor activity of T lymphocytes. Our data map the route of a clinically feasible strategy to promote the antitumor immune responses elicited by combined ICB, while preventing autoimmune reactions

To validate the results obtained in the mouse models and further probe the connection of TNF with ICB efficacy, we next performed an mRNA analysis of immune-related genes from biopsies of i) healthy mucosal tissue in cancer patients, ii) colon tissue from patients diagnosed with colitis after nivolumab and/or ipilimumab administration or iii) colon tissue from patients diagnosed with ulcerative colitis. In both ICB-mediated colitis and ulcerative colitis, *TNF* mRNA was significantly upregulated when compared to colon healthy tissue samples. In addition, a gene signature of TNF active tray was present in the mucosa of these patients. Using a xenograft-versus-host model disease, in which human peripheral blood mononuclear cells (PBMCs) were injected intravenously into Rag2^-/-^IL2rg^-/-^ mice, we observed that ipilimumab and nivolumab treatment exacerbated immune-mediated colitis with an increase of weight loss and inflammation of the large intestine signified by the thickness of the intestinal wall. As expected from results obtained in mouse models, this effect was minimized by the co-treatment with etanercept. Moreover, in the presence of the human tumor cell line HT29 in the xenograft-versus-host model disease, etanercept treatment retained the ICB-mediated anti-tumor efficacy and decreased the level of alanine transaminase and aspartate transaminase in serum as well as reduced the thickness of the intestinal wall, all of which suggest a reduction in hepatitis and colitis that result from the xenograft from the host reaction. These results recapitulated the data obtained in mouse models and provide evidence for the potential benefits of using TNF blockade therapy to ameliorate the ICB therapy-dependent irAEs.

## CONCLUSION

Despite the enormous efforts made to improve anti-tumor responses by ICB therapy, the manifestation of treatment-related toxicities has become a critical limiting factor for its applicability. Thus, efforts towards improving tolerability are required. Taking into consideration our findings, the administration of TNF neutralization agents prophylactically or concomitantly with ICB therapy was capable of resolving these shortcomings for ICB immunotherapy, while retaining its anti-tumor efficacy, presenting a strategy that allows for a separation between efficacy and toxicity (**Figure 1**). Moreover, with this approach, a sustained and/or higher dose regimen of ICB compounds would be tolerated, which could strengthen its anticarcinogenic potential. Currently, a phase I investigator-initiated trial (https://clinicaltrials.gov; NCT03293784) is undergoing to evaluate the safety of the proposed approach. Sufficiently powered randomized clinical trials measuring overall response rate and the percentage of irAEs > grade 3 are the pathway forward.

